# Identification and characterization of structural variants related to meat quality in pigs using chromosome-level genome assemblies

**DOI:** 10.1186/s12864-024-10225-1

**Published:** 2024-03-21

**Authors:** Daehong Kwon, Nayoung Park, Suyeon Wy, Daehwan Lee, Woncheoul Park, Han-Ha Chai, In-Cheol Cho, Jongin Lee, Kisang Kwon, Heesun Kim, Youngbeen Moon, Juyeon Kim, Jaebum Kim

**Affiliations:** 1 Department of Biomedical Science and Engineering, Konkuk University, Seoul, 05029 Republic of Korea; 2 Animal Genomics and Bioinformatics Division, National Institute of Animal Science, RDA, Wanju, 55365 Republic of Korea; 3 Subtropical Livestock Research Institute, National Institute of Animal Science, RDA, Jeju, 63242 Republic of Korea

**Keywords:** Meat quality, Structural variation, Nanchukmacdon, Chromosome-level genome assembly, Multi-omics analysis

## Abstract

**Background:**

Many studies have been performed to identify various genomic loci and genes associated with the meat quality in pigs. However, the full genetic architecture of the trait still remains unclear in part because of the lack of accurate identification of related structural variations (SVs) which resulted from the shortage of target breeds, the limitations of sequencing data, and the incompleteness of genome assemblies. The recent generation of a new pig breed with superior meat quality, called Nanchukmacdon, and its chromosome-level genome assembly (the NCMD assembly) has provided new opportunities.

**Results:**

By applying assembly-based SV calling approaches to various genome assemblies of pigs including Nanchukmacdon, the impact of SVs on meat quality was investigated. Especially, by checking the commonality of SVs with other pig breeds, a total of 13,819 Nanchukmacdon-specific SVs (NSVs) were identified, which have a potential effect on the unique meat quality of Nanchukmacdon. The regulatory potentials of NSVs for the expression of nearby genes were further examined using transcriptome- and epigenome-based analyses in different tissues.

**Conclusions:**

Whole-genome comparisons based on chromosome-level genome assemblies have led to the discovery of SVs affecting meat quality in pigs, and their regulatory potentials were analyzed. The identified NSVs will provide new insights regarding genetic architectures underlying the meat quality in pigs. Finally, this study confirms the utility of chromosome-level genome assemblies and multi-omics analysis to enhance the understanding of unique phenotypes.

**Supplementary Information:**

The online version contains supplementary material available at 10.1186/s12864-024-10225-1.

## Background

Meat quality has been an important feature in the livestock industry throughout human history. The importance of the trait has increased in the last 50 years with the increased demand for high-quality meats, and the trait is currently a considerably important characteristic in a related industry [1, 2]. Therefore, the livestock industry has continuously developed new breeds that can provide higher-quality meat to meet the demands of customers [3]. As the genomic sequences and annotations of many pig breeds have been revealed [4], it is important to understand the genetic architectures regulating the trait to make a new breed with better meat quality. Based on the maturity of sequencing technologies, diverse genomic studies have been performed to identify genomic loci affecting meat quality in pigs. Especially, several population-level studies using short read data, such as population genomic analyses [5–7] and genome-wide association studies [8–10], have identified plentiful regions and genes associated with the traits at the whole-genome level.

However, it is still not enough to fully uncover the genetic architectures for the trait because the previous studies mainly focused on small variants such as single nucleotide polymorphisms, small insertions and deletions. Structural variations (SVs), which are large variants involving more than 50 base pairs, also contribute to phenotypic alterations [11–13]. However, understanding the effect of SVs on meat quality in pigs has lagged because it has been hard to accurately discover SVs due to the limited availability of sequencing data and the incompleteness of genome assemblies especially for a pig breed specifically inbred for higher meat quality [14, 15]. In this situation, the high-quality and chromosome-level genome assemblies of such pig breeds can be used as valuable resources to accurately identify SVs [16, 17] even in repetitive and complex regions [18].

We previously reconstructed a chromosome-level genome assembly [19], called the NCMD assembly, for Nanchukmacdon which is a new pig breed derived by mating three different commercial pig breeds (Korean native pig, Duroc and Landrace) with outstanding levels of intramuscular fat deposition and redness in meat compared to other pig breeds [19, 20]. Therefore, the high-quality and chromosome-level NCMD assembly can provide new opportunities for fully understanding the impact of SVs on meat quality in pigs.

In this study, we discovered SVs in diverse pig breeds including Nanchukmacdon by comparing the prebuilt NCMD assembly against different pig genome assemblies. Furthermore, we identified several candidate Nanchukmacdon-specific SVs (NSVs) responsible for the superior meat quality of Nanchukmacdon. In addition, the analysis using multi-omics data enabled us to interrogate the regulatory roles of the NSVs for the expression of adjacent genes related to meat quality. This study will establish a foundation for understanding the role of SVs on breed-specific traits, and act as an important resource for future development of breeds with superior meat quality.

## Methods

### Collinearity comparison of the chromosome-level assemblies of pig breeds

For comparing genome assemblies of pig breeds, the genome assemblies of the Nanchukmacdon (NCMD), Duroc (Sscrofa11.1) and Meishan (MSCAAS v1) were obtained from the NCBI database (accession GCA_031306245.1, GCF_000003025.6, and GCA_017957985.1). Whole-genome sequence alignments of the NCMD assembly against the MSCAAS v1 and Sscrofa11.1 (the Duroc pig) assembly were next constructed using LASTZ (v.1.04.00) [21]. Synteny blocks among them were then constructed by the synteny block detection program in InferCars [22] with a resolution of 300 Kbp. Breakpoint regions, which are genomic regions between two adjacent genomic blocks belonging to different synteny blocks, were identified in each assembly using the in-house script. The syntenic relationships and breakpoint regions between the two assemblies were visualized using mySyntenyPortal [23] and jcvi (https://github.com/tanghaibao/jcvi).

For analyzing the mapping patterns of the sequencing reads at the breakpoint regions, we obtained long and short reads of Nanchukmacdon and Meishan from the previous studies [19, 24]. The short and long reads were first mapped against the corresponding assembly (the NCMD assembly for the reads of Nanchukmacdon, and the MSCAAS v1 assembly for the reads of Meishan). Then, the reads mapped to the breakpoint regions of each assembly were next mapped to the other two assemblies of different pig breeds. The short and long reads were mapped using BWA MEM [25] and minimap2 (v.2.17) [26], respectively. Also. the Hi-C reads of Nanchukmacdon were also obtained from a recent study [19] and aligned to the other two assemblies, and the Hi-C contact maps at the breakpoint regions in each assembly were constructed using the HiC-Pro pipeline (v.2.11.4) [27] with default parameters and 100 Kbp bins. The Hi-C contact maps were visualized using HiCPlotter (v.0.8.1) [28].

To assess the functional effect of genome rearrangements, the breakpoint regions were annotated using SnpEff (v.4.3t) [29] with “-ud 5000” parameter. For the annotation of breakpoint regions, we used the pig reference (Sscrofa11.1.101) and Nanchukmacdon gene annotation information which were downloaded from the NCBI database and a previous paper [19], respectively. We defined the breakpoint regions affecting exons or 3/5 prime UTRs as exonic breakpoint regions.

### Variant analysis

To call variants based on genome assemblies, whole-genome pairwise alignment was performed for each of the NCMD and Landrace_pig_v1 assembly (Landrace pig) against the Sscrofa11.1 genome assembly (Duroc pig) using minimap2 (v.2.17) with "-a -x asm5" parameters. For that, the public genome assembly of Landrace was additionally obtained from the NCBI database (GCA_001700215.1). Using the alignment results, structural variants (SVs) were called in each assembly with SVIM-asm (v.1.0.3) [30]. We excluded the breakend variants in this study. To identify candidate Nanchukmacdon-specific variants (NSVs), we filtered out the variants found in the NCMD assembly whose positions on the Sscrofa11.1 assembly overlapped those found in the Landrace_pig_v1 assembly. The called NSVs were annotated using VEP [31] with the pig reference annotation information (Sscrofa11.1.101) downloaded from NCBI database. We defined the stop gained/lost, frameshift variant, inframe insertion/deletion, coding sequence variant, and 3/5 prime UTR variant as exonic variants, splice donor/acceptor/region variant and intron variant as intronic variants, and upstream/downstream gene variant and intergenic variant as intergenic variants. GO enrichment analysis for the NSVs was conducted using g:Profiler [32] with g:SCS threshold 0.05.

### NSV genotyping analysis

To genotype NSVs, we obtained the whole genome sequencing data of 10, 6, and 13 samples of Nanchukmacdon, Duroc, and Landrace, respectively, from the NCBI database (Additional file 2: Table S1). The quality of the collected whole genome sequencing data was examined using FastQC (v.0.11.9) [33]. Low-quality reads and adaptor sequences in the reads were removed using NGStoolkit IlluQC.pl (v.2.3.3) [34]. When there were no adapter sequences in the reads, “N A -s 20 -l 70” parameters were used for NGStoolkit to filter only low-quality reads with low-quality base percentages (Phred quality score < 20) larger than 30%. If adapter sequences were present, “2 A -s 20 -l 70” parameters were used. Low-quality bases (Phred quality score < 20) were next trimmed at the 3' end of the reads, and the trimmed reads shorter than 45 bases were removed using NGStoolkit TrimmingReads.pl with “-q 20 -n 45” parameters. If adapter sequences still remained when checked with FastQC, additional trimming was performed using TrimGalore (v.0.6.0) [35] with the following parameters: “-q 20 –length 45 –paired –illumina”. The cleaned reads for each sample were mapped against the pig reference assembly (Sscrofa11.1) using BWA MEM (v.0.7.17) [25]. The NSVs are genotyped using the mapping results with graphtyper (v.2.7.5) [36]. We only used the aggregated genotype calls and filtered out the calls not annotated as PASS for further analysis.

### PCA analysis

Based on the genotyped NSVs in autosomes, a principal component analysis (PCA) was performed using the Genome-wide Complex Trait Analysis (GCTA) tool (v.1.91.4) [37]. The genetic relationship matrix for pairs of individuals was estimated using “–make-grm –autosome –autosome-num 18” parameters. Based on the matrix, eigenvalues and eigenvectors were calculated using the “–pca 3” parameter in GCTA. The results were visualized using the ggplot2 R package [38].

### Integrative multi-omics analysis for NSV flanking genes

For the differential gene expression (DGE) analysis, we first collected the raw RNA sequencing (RNA-seq) data from 15, 12, and 17 samples of three different pig breeds for adipose, liver and muscle tissues, respectively. The raw RNA-seq data of the three tissues of Nanchukmacdon were obtained from previous studies [20, 39]. The raw RNA-seq data of the other two pig breeds (Duroc and Landrace) were obtained from the NCBI database (Additional file 2: Table S2). For each tissue, a separate DGE analysis was performed for Nanchukmacdon against each of Duroc and Landrace using the RNA-seq data. The DGE analyses were performed by following the procedure in a previous study [20]. In brief, the quality of RNA-seq reads was checked using FastQC [40], and they were trimmed by Trimmomatic [41]. The trimmed RNA-seq reads of each pig breed were aligned to the pig reference assembly (Sscrofa11.1) using HISAT2 [42] with default parameters. For each gene in the pig reference assembly, the number of mapped RNA-seq reads was counted using FeatureCount [43], and the DGE analysis between two pig breeds was performed using the DESeq2 R package [44]. Genes with |log2FC|> = 1 and an adjusted *P*-value < 0.05 were identified as the final differentially expressed genes (DEGs). Finally, we identified the common DEGs of Nanchukmacdon against the two breeds for each tissue.

For the epigenome analysis, we obtained the H3K4me1, H3K27ac and H3K4me3 histone modification profile and chromatin state information of pigs from a previous study [45]. For visualization, we used histone modification profile data of the P348 sample.

## Results

### Collinearity comparison with chromosome-level assemblies of pig breeds

**Fig. 1:**
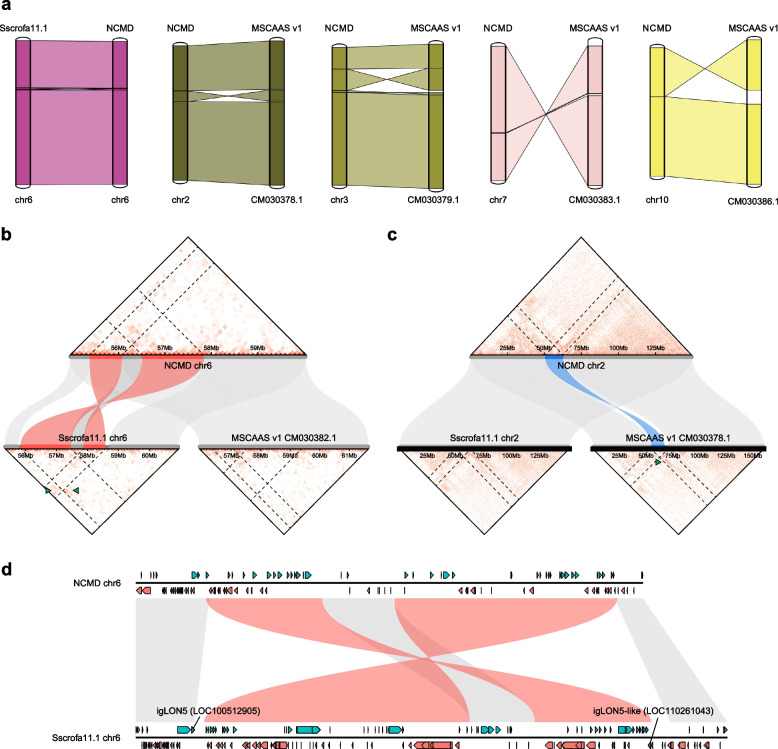
Comparison of the NCMD assembly with assemblies of other pig breeds. **a** Syntenic relationships of Nanchukmacdon chromosomes containing breakpoint regions against the Duroc (Sscrofa11.1) and Meishan (MSCAAS v1) chromosomes. The syntenic relationships were visualized using mySyntenyPortal (http://bioinfo.konkuk.ac.kr/mySyntenyPortal_RDA/publish/Nanchukmacdon_2Breed). **b, c** Examples of breakpoint regions in the Nanchukmacdon genome against Meishan (**b**) and Duroc (**c**) genome with Hi-C heatmaps in the regions. Colored regions in each assembly represent the regions corresponding to the breakpoint regions in the assembly. Green triangles pinpoint irregular Hi-C contact frequencies near breakpoint regions. **d** Paralogous genes flanking in the breakpoint regions between the Nanchukmacdon and Duroc genome. Green and red boxes respectively represent the genes in forward and reverse strand in each genome, respectively

We first compared the NCMD assembly against chromosome-level assemblies of two different pig breeds, such as MSCAAS v1 for Meishan and Sscrofa11.1 for Duroc by constructing synteny. The compared assemblies were overall collinear with only four and eight breakpoint regions in the NCMD assembly against the Sscrofa11.1 and MSCAAS v1 assembly, respectively (Fig. 1a).

To check whether the identified breakpoint regions are the results of misassembly in the NCMD assembly or not, the Hi-C contact maps were created by mapping the Hi-C reads of Nanchukmacdon to each of the assemblies, and the generated Hi-C contact maps were compared. As shown in Fig. 1b-c, normal contact frequencies were observed in all breakpoint regions in the NCMD assembly, while abnormal patterns were found in the corresponding breakpoint regions in the other two assemblies. Mapping patterns of the short and long Nanchukmacdon reads against the three assemblies were also investigated (Additional file 1: Figure S1-S5). Both short and long reads were properly mapped in the breakpoint regions of the NCMD assembly only, but not in those of the other two assemblies. Consistent patterns were also observed when the reads of Meishan were mapped (Additional file 1: Figure S6). These results indicate that the observed genome rearrangements are not artifacts generated by the misassembly of the NCMD assembly.

Six and 35 genes were located within 5 Kbp upstream and downstream of the breakpoint regions against Duroc and Meishan, respectively (Additional file 2: Table S3-S4). Most of the breakpoints were in intronic and intergenic regions, and only one (1.45%) and eight (5.00%) exonic regions were affected by the breakpoint regions against Duroc and Meishan, respectively (Additional file 2: Table S5). Interestingly, some of these genes were paralogous to each other. For example, the igLON Family Member 5 (igLON5; LOC100512905) and igLON5-like (LOC110261043) gene were located close to the boundary of breakpoint regions against Duroc (Fig. 1d). The breakpoint region against Meishan also contained several olfactory receptor genes (Additional file 1: Figure S7).

### Identification of structural variants related to Nanchukmacdon-specific phenotypes

**Fig. 2:**
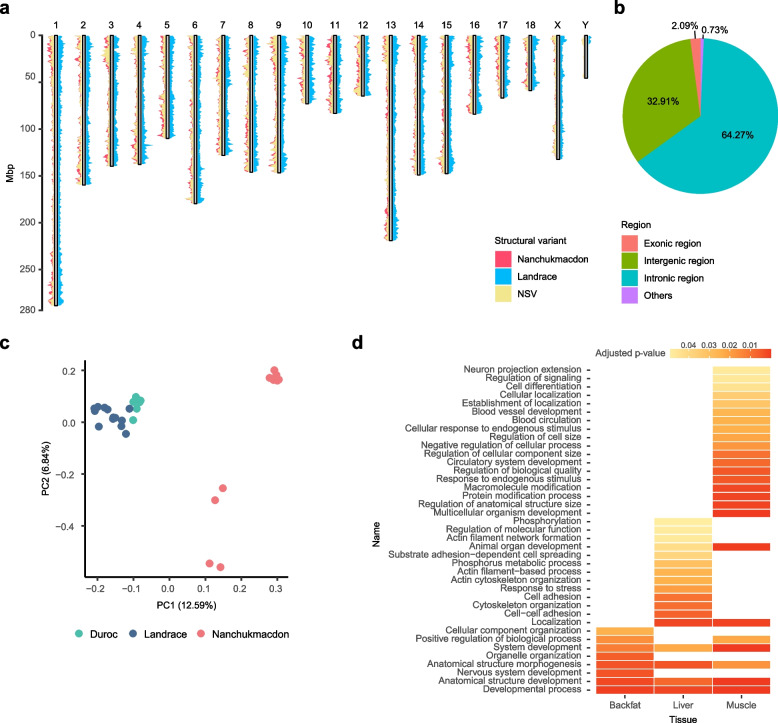
Structural variants (SVs) in Nanchukmacdon and Landrace. **a** Distribution of SVs in the Nanchukmacdon and Landrace genomes. NSVs represent Nanchukmacdon-specific SVs. **b** Annotation result for NSVs. **c** PCA result of 29 samples of three different pig breeds based on NSVs. **d** Enriched biological process gene ontology terms for differentially expressed genes related to NSVs in the backfat, liver, and muscle tissue

We also used the NCMD assembly to identify genomic features related to the superior phenotype of Nanchukmacdon compared to its related breeds (Duroc, Landrace) including higher levels of intramuscular fat deposition and reddish meat color [20]. Here, putative structural variants (SVs) of Nanchukmacdon and Landrace were identified against Duroc based on whole-genome pairwise comparisons (Methods). A total of 30,784 and 62,373 of SVs in length $\ge$ 50 bp were detected in the genome of Nanchukmacdon and Landrace, respectively (Table 1). Most of the SVs were insertions or deletions in both breeds. As described in the previous genomic variant studies [46, 47], the SVs were not randomly distributed in both breeds, and more SVs were observed in the telomere regions in both breeds (Fig. 2a).

**Table 1:** Statistics of structural variants in Nanchukmacdon and Landrace genome. NSV: Nanchukmacdon-specific variant, DEL: Deletion, INS: Insertion, DUP: Duplication, INV: Inversion

Breed	Type	Total number	Median length (bp)	Total length (bp)	No. of NSVs
Nanchukmacdon	DEL	15,636	288	12,662,862	7,012
	DUP	96	5,297	859,163	70
	INS	14,986	281	7,038,645	9,785
	INV	66	1,576.5	347,275	31
Landrace	DEL	28,573	151	10,049,685	-
	DUP	343	752	511,567	-
	INS	33,361	127	7,912,026	-
	INV	96	1,161	798,021	-

To determine the SVs affecting the superior phenotypes of Nanchukmacdon, we found the candidate Nanchukmacdon-specific SVs (NSVs), which were observed only in the Nanchukmacdon genome (Method). We identified 16,898 NSVs including 7,012 deletions, 70 duplications, 9,785 insertions, and 31 inversions (Fig. 2a, Table 1). The NSVs were genotyped using whole genome sequencing data of total 29 samples of Nanchukmacdon, Duroc, Landrace (Additional file 2: Table S6). An average of 7,051 (41.73%) NSVs were genotyped in each sample, and the number of genotyped NSVs of Nanchukmacdon was larger than those of the other breeds. In PCA performed using the genotyped NSVs in autosomes, clear separation between Nanchukmacdon and the other breeds was identified by the first PC (Fig. 2c). To further identify the separation came from the differences of the rate of genotyped NSVs among breeds, we performed the PCA analysis using the NSVs with minor allele frequency not lower than 0.05 and missing rate not larger than 0.1. The clear division between Nanchukmacdon and the other breeds was consistently identified in the PCA using the filtered NSVs (Additional file 1: Figure S8).

When annotating those NSVs, a total of 4,793 genes contained the NSVs within 5 Kbp upstream and downstream of their gene body. We also found that only 2.09% (734) NSVs were mapped within exonic regions. The remaining 64.27% (22,613) and 32.91% (11,581) were in intronic and intergenic regions, respectively (Fig. 2b, Additional file 2: Table S7). For the genes adjacent to or containing the NSVs, differential gene expression analyses were followed. In this analysis, 414, 473 and 558 genes were differentially expressed only in Nanchukmacdon (not both in Duroc and Landrace) in the backfat, liver and muscle tissue of Nanchukmacdon, respectively (Additional file 2: Table S8).

For those differentially expressed genes (DEGs) in each tissue, the gene ontology (GO) enrichment analysis was performed (Additional file 2: Table S9). For biological process terms, the muscle and backfat tissue had the highest and lowest number of enriched terms, respectively (Fig. 2d). In the backfat tissue, the DEGs were mainly involved in several development-related terms, such as developmental process (GO:0032502), anatomical structure development (GO:0048856) and system development (GO:0048731). Interestingly, most of the development-related terms were commonly identified in all three tissues. In addition, we also identified many tissue-specific enriched GO terms in liver and muscle, such as cell adhesion (Cell adhesion (GO:0007155), cell–cell adhesion (GO:0098609)), actin filament (Actin filament-based process (GO:0030029), actin cytoskeleton organization (GO:0030036), actin filament network formation (GO:0051639)), and phosphorylation process (Phosphorylation (GO:0016310), phosphorus metabolic process (GO:0006793)) for liver, and macromolecule modification (Macromolecule modification (GO:0043412), protein modification process (GO:0036211)), cell development (Cell differentiation (GO:0030154), regulation of cell size (GO:0008361), regulation of anatomical structure size (GO:0090066), regulation of cellular component size (GO:0032535)) and blood circulatory system development (Circulatory system development (GO:0072359), blood circulation (GO:0008015), and blood vessel development (GO:0001568)) for muscle. These results suggest that the NSVs can also shape the superior phenotypes of Nanchukmacdon in a tissue-specific manner.

### Regulatory potentials of NSVs affecting the expression of nearby genes

**Fig. 3:**
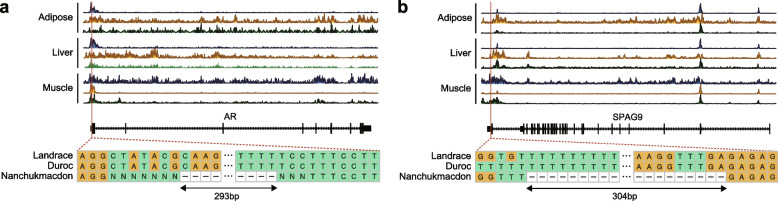
Example NSVs potentially affecting expression of the AR (**a**) and SPAG9 (**b**) gene. Red colored lines highlight the location of NSVs. Area charts with blue, yellow and green color indicate H3K4me1, H3K4me3, and H3K27ac signals of corresponding regions respectively

Using chromatin state data obtained from a published paper [45], we examined the regulatory potentials of NSVs affecting the expression of nearby genes related to the specific phenotypes of Nanchukmacdon. We found that 643 NSVs near DEGs were located in actual regulatory regions (Additional file 2: Table S10). For example, an NSV with a length of 293 bp (Deletion at Sscrofa11.1: X:53,609,703–53,609,995) was discovered in the first intron of the androgen receptor (AR) gene which is involved in the diverse developmental terms (Fig. 3a). This NSV was mapped flanking to the active promoter region (TssAFlnk) in all three tissues (Additional file 2: Table S10). Also, as shown in Fig. 3b, strong signals were observed from all three histone modification marks containing the H3K4me3 signal, an indicator of the active promoter, in all three tissues. The RNA-seq data revealed that the AR gene was commonly down-regulated in all three tissues only in Nanchukmacdon, not in the other two breeds. These results indicate that the deletion could inhibit the AR gene expression by disrupting promoter activity.

We also identified an NSV with a length of 304 bp (Deletion at Sscrofa11.1:12:27,201,206–27,201,509) in the last exon of the sperm associated antigen 9 (SPAG9) gene which was down-regulated only in the muscle tissue of Nanchukmacdon (Fig. 3b). The NSV was located in the enhancer region (EnhG) with relatively high H3K4me1 and H3K27ac signal only in the muscle tissue (Fig. 3b, Additional file 2: Table S10). Because those marks are indicators of an active enhancer, the deletion in that region by the NSV could prevent a normal expression of the SPAG9 gene only in the muscle tissue of Nanchukmacdon. The genotypes of both NSVs were not fully identified in the three breeds.

## Discussion

In this study, we investigated the role of SVs on the meat quality trait by comparing the genomes of Nanchukmacdon and other pig breeds. Nanchukmacdon breed shows various unique meat quality phenotypes compared to other commercial pig breeds. Especially, the breed shows outstanding levels of intramuscular fat feature which is one of the most important factors determining the quality of meat [48]. Moreover, the recent construction of a chromosome-level genome assembly of Nanchukmacdon, called the NCMD assembly [19], has provided the full genomic structure of the breed. The high-quality genome assembly enables to accurately identify large and complex SVs [16, 17]. These make Nanchukmacdon a good animal model for studying the impacts of SVs on meat quality.

Here, using the NCMD assembly, we confirmed two different scales of SVs within pig species and the related biological features. For example, we found several large-scale genomic rearrangements among pig breeds through the collinearity analysis of chromosome-level assemblies based on the synteny. Especially, even though Duroc genome assembly was used to construct the NCMD assembly, we successfully revealed the rearrangement events between Nanchukmacdon and Duroc. In addition, we identified that there are several paralogous genes, such as igLON, close to the breakpoint regions. It suggests that the non-allelic homologous recombination (NAHR) between paralogous genes is one of the mechanisms of genomic rearrangements in pig species. Also, we identified 30,784 SVs including deletions, duplications, insertions and inversions in the Nanchukmacdon genome against Duroc pig. In particular, we discovered 16,898 NSVs that may contribute to the unique meat quality phenotypes of the Nanchukmacdon breed through comparison with the SVs of the Landrace breed.

Furthermore, the impacts of NSVs on the phenotypes were further interrogated with diverse multi-omics data. We identified the 414, 473 and 558 DEGs containing or close to the NSVs in the backfat, liver and muscle tissue of Nanchukmacdon against the European domestic pigs. Especially, these DEGs were related to several development-related GO terms in all three tissues. The development-related terms have been reported to affect intramuscular fat (IMF) traits in many previous studies [49–51]. We also identified the terms that appeared in a specific tissue, for example, cell adhesion, actin filament, and phosphorylation process for liver, and macromolecular modification, cell and blood circulation system development for muscle tissue. The actin filaments perform diverse cellular functions including cell adhesion and directly or indirectly regulate lipid synthesis and metabolism [52]. Also, the phosphorylation has been reported to be involved in the hepatic lipid metabolism [53]. The blood circulation also has been reported to affect IMF traits in chickens [54]. Interestingly, while lipid biosynthesis mainly occurs in the adipose tissue of pigs, lipids are mainly synthesized in liver tissue and transported via blood circulation in chickens [54, 55]. The significant GO terms related to lipid metabolism in liver and blood circulation in muscle suggest that the lipid biosynthesis mechanism like chickens can exist in Nanchukmacdon and affect its high IMF content. The cell development-related terms also seem to be involved in the superior meat quality, such as reddish color [56].

We also found the impact of NSVs on the expression of flanking genes investigated with the pig histone modification profile data and confirmed that several NSVs have functional regulatory effects on flanking genes, for example AR and SPAG9. The AR gene involved in the diverse development terms is commonly down-regulated in the all three tissues of Nanchukmacdon compared to the different pig breeds. The AR gene generates the Androgen receptor protein (AR), and the AR signaling pathway regulates the body fat mass [57, 58] and muscle development [59, 60]. Especially, the AR-knockout mouse models show lipogenesis and increased fat mass [60]. In addition, the sex hormones including androgen, which are ligands of the AR protein, are also reported to be associated with the amount and distribution of body fat [57, 61, 62]. SPAG9 promotes the MAPK signaling pathway which regulates lipid deposition and metabolism [63–65]. Also, it interacts with the NRP1 gene involved in adipogenesis [66]. We suggest that the NSVs in these genes could shape the outstanding levels of intramuscular fat deposition and redness in meat of Nanchukmacdon by affecting expression of their nearby genes.

Unfortunately, the genotyping of the NSVs using short reads was not possible in this study, which may be caused by the limitation of short reads for genotyping [67]. In addition, the specificity of NSVs on Nanchukmacdon was not experimentally validated. Thus, the genotyping using other types of sequencing data, such as long reads, and the experimental validation of NSVs by PCR or gene expression assays will be performed as a future research direction.

## Conclusion

In this study, we performed the assembly-based variant calling using the chromosome-level genome assemblies of multiple pig breeds, and identified various SVs and their diverse biological features related to genome structure and rearrangements in pigs. Furthermore, the potential SVs shaping the unique meat phenotypes of Nanchukmacdon, called NSVs, were detected through comparative analysis of SVs among different pig breeds. The impact of the NSVs on the meat quality trait was further investigated by analyses using diverse multi-omics data. These findings contribute to a better understanding of the biological mechanisms of SVs underlying the meat quality trait in pigs. Also, integrative analyses of the assembly-based variant calling with diverse multi-omics data show the great potential to reveal SVs related to unique and breed-specific phenotypes.

### Supplementary Information

**Additional file 1. **Supplementary Figures S1-S8

**Additional file 2. **Supplemental Tables S1-S10

## Data Availability

For comparing genome assemblies of different pig breeds, the genome assembly of the Meishan pig (MSCAAS v1) was obtained from the NCBI database (accession GCA_017957985.1). The public genome assembly of Duroc and Landrace was obtained from the NCBI database (accession GCF_000003025.6 and GCA_001700215.1, respectively), and the chromosome-level NCMD assembly was obtained from a recent study [16]. The called NSVs were annotated using VEP [27] with the pig reference annotation information (Sscrofa11.1.101) downloaded from NCBI database.

## Abbreviations

SV: Structural variant
NSV: Nanchukmacdon-specific structural variant
log2FC: Log_2_ fold change
IMF: Intramuscular fat
DEG: Differentially expressed gene
DGE analysis: Differential gene expression analysis
NAHR: Non-allelic homologous recombination
