# Supplementary material for: Identification and characterization of structural variants related to meat quality in pigs using chromosome-level genome assemblies
Source: BMC Genomics. 2024 Mar 21;25:299. doi: 10.1186/s12864-024-10225-1 (PMC10956321; doi:10.1186/s12864-024-10225-1)
Supplement: Supplementary file 1 — Additional file 1. Supplementary Figures S1-S8 [file 12864_2024_10225_MOESM1_ESM.docx]

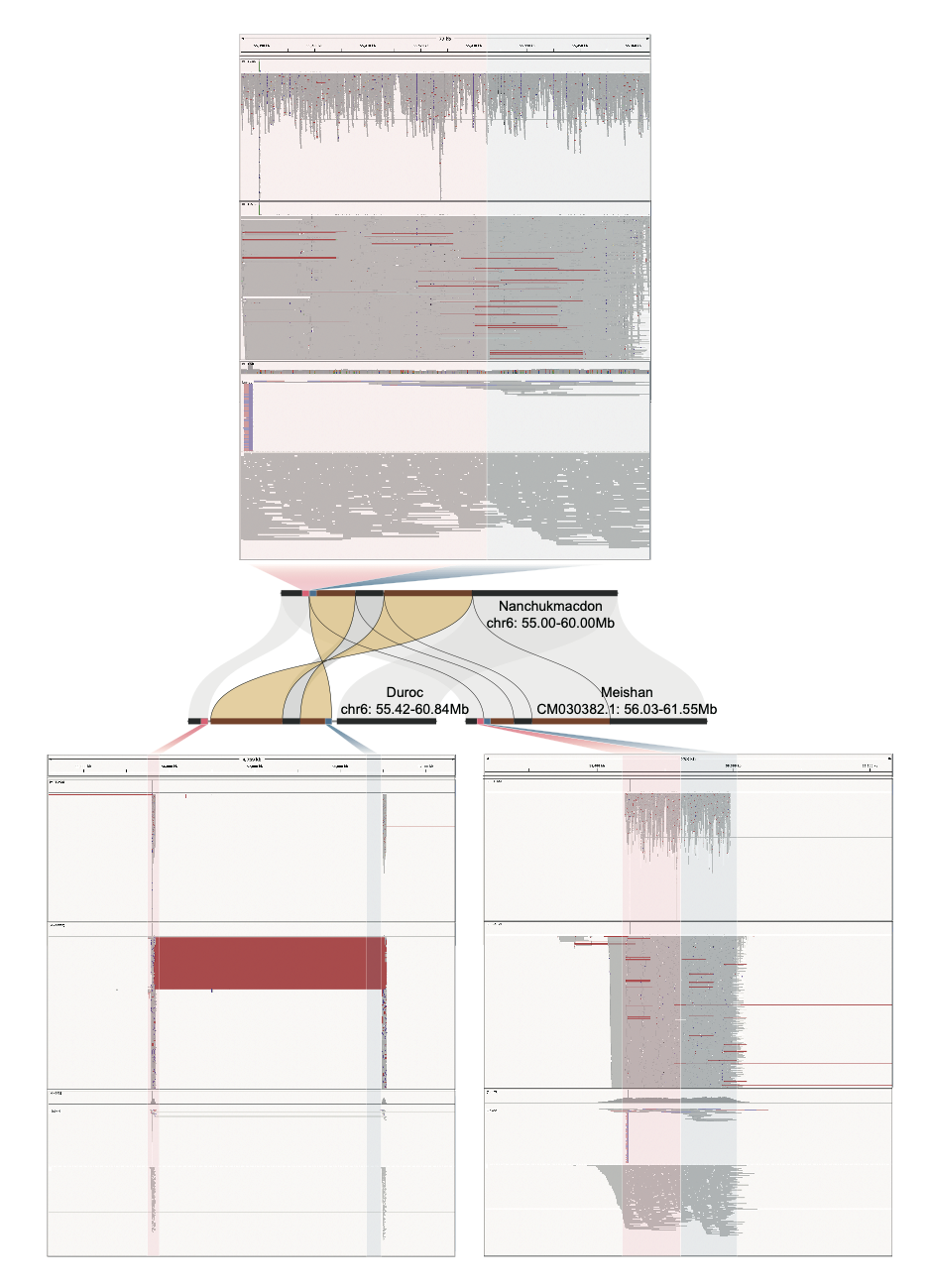


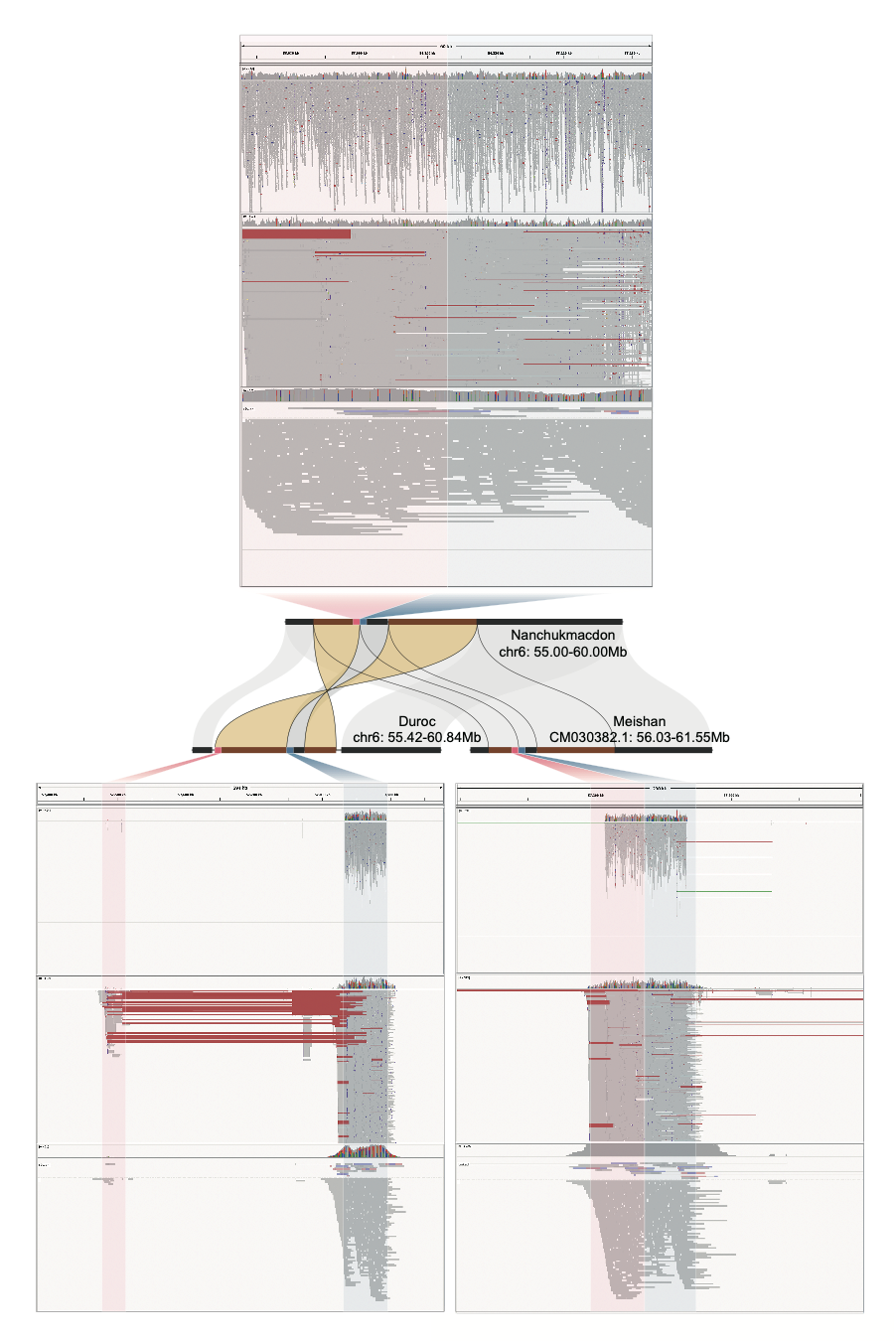


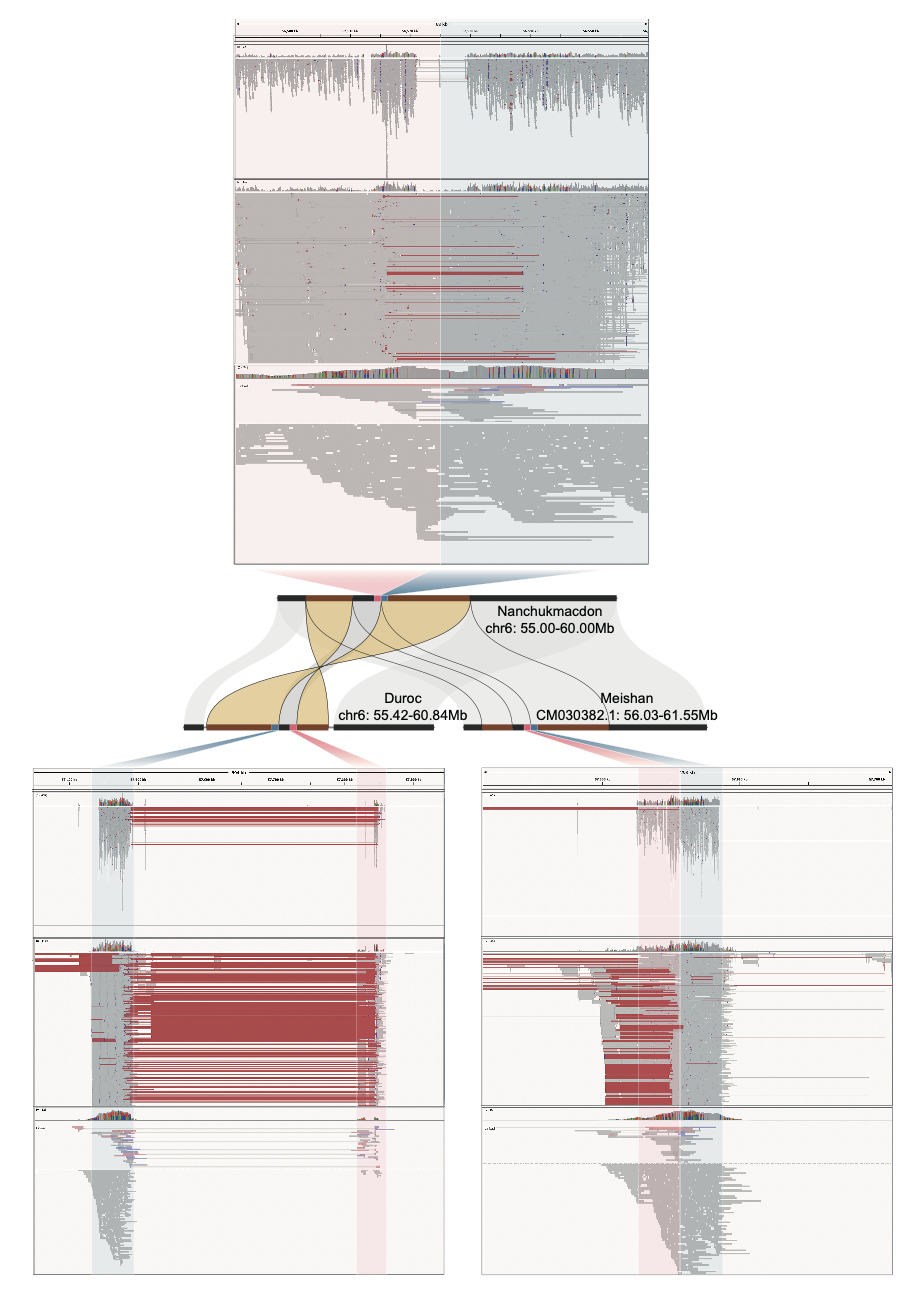


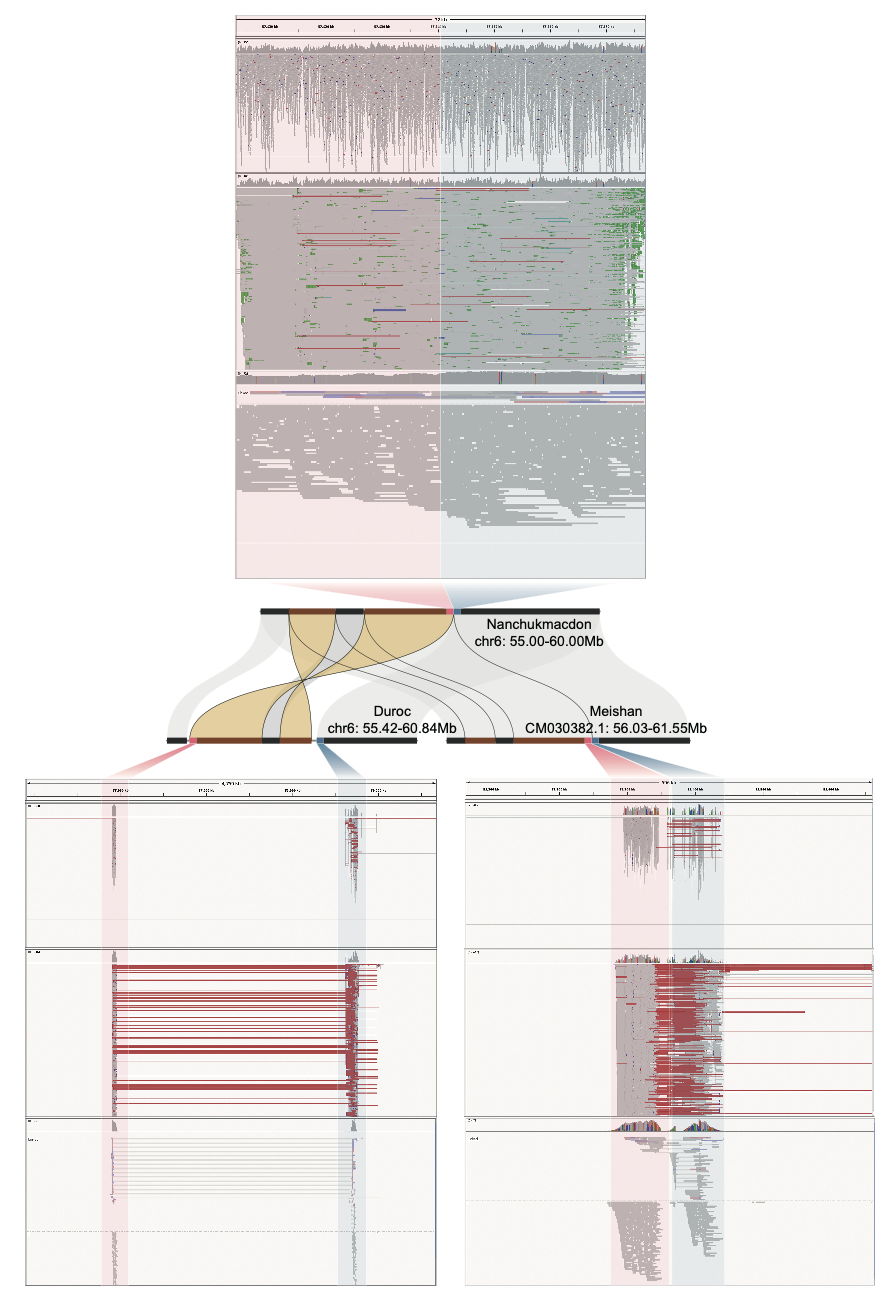


**Figure S1.** Mapping patterns of Nanchukmacdon reads at the breakpoint regions of different pig breed assemblies in chromosome 6. The panels show the mapping depths and patterns of paired-end (Top), mate pair (Middle), and long reads (Bottom) at the breakpoint regions of each breed, respectively. The reads which were mapped with mapping quality larger than 10 were only shown in the figure. The grey and red lines in short read panels represent the reads mapped with proper insert size and not. The grey lines in long read panels represent read alignments and links between primary and supplementary alignments of the same read. The read mapping patterns were drawn using IGV program.


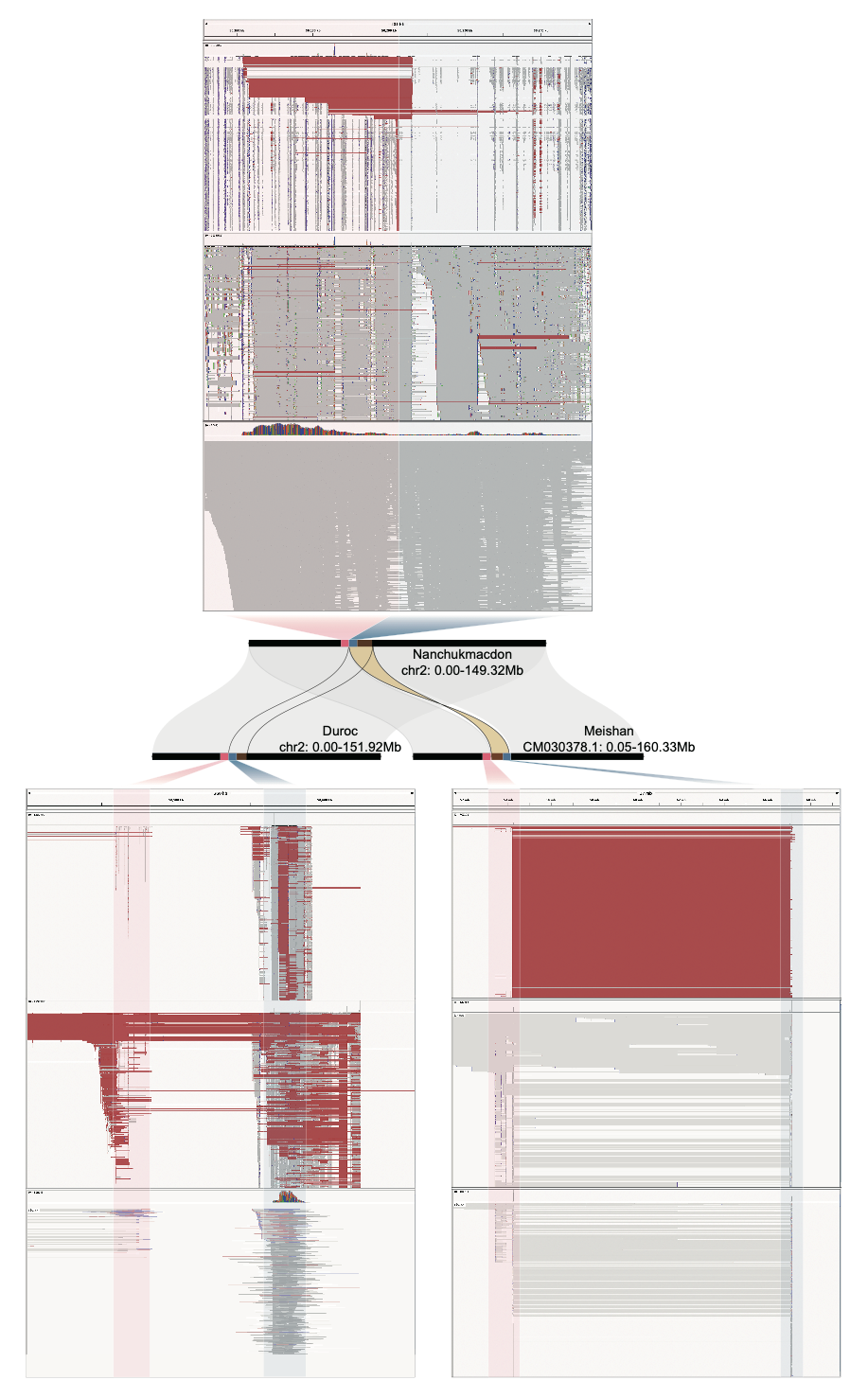


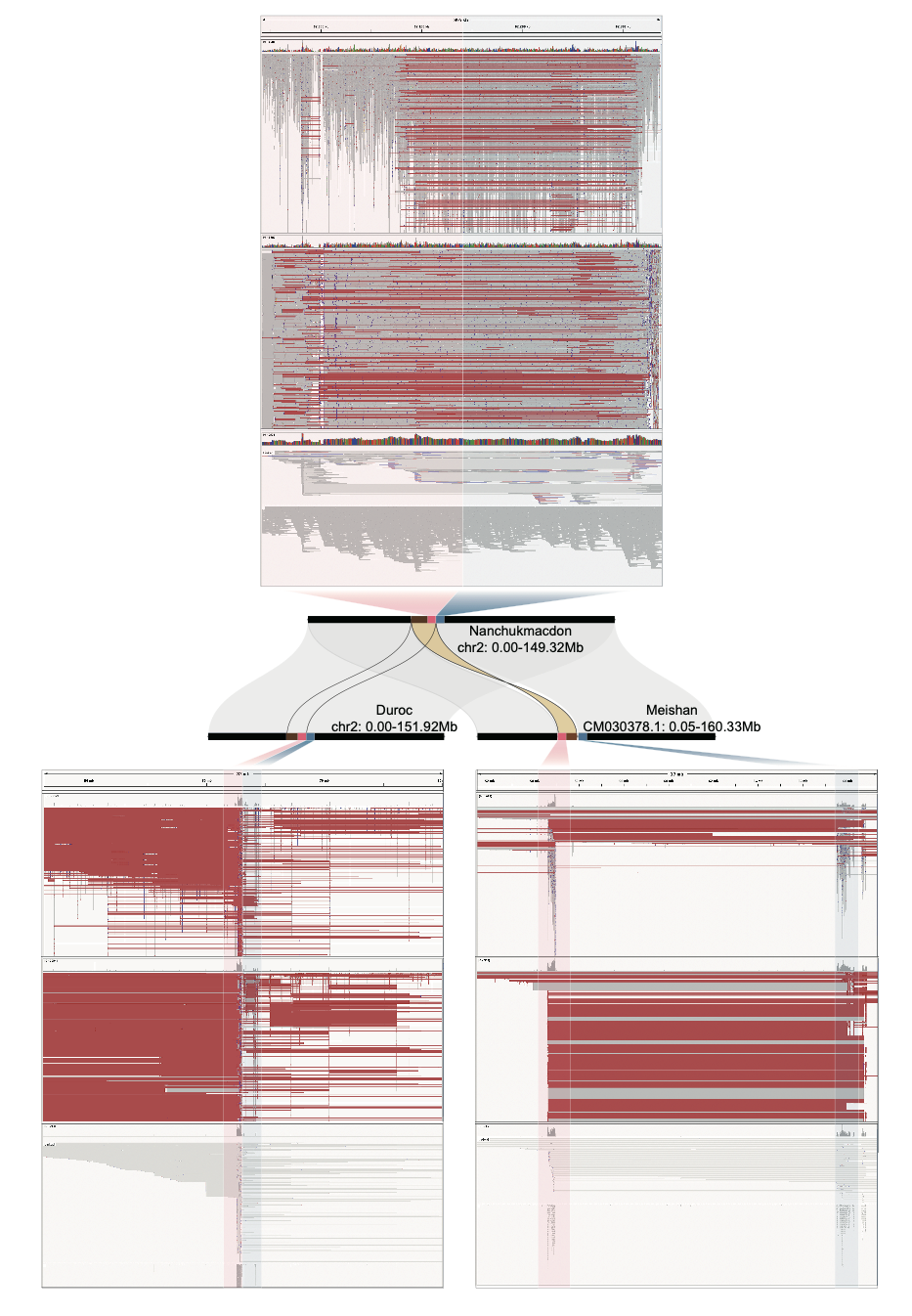


**Figure S2.** Mapping patterns of Nanchukmacdon reads at the breakpoint regions of different pig breed assemblies in chromosome 2. The panels show the mapping depths and patterns of paired-end (Top), mate pair (Middle), and long reads (Bottom) at the breakpoint regions of each breed, respectively. The reads which were mapped with mapping quality larger than 10 were only shown in the figure. The grey and red lines in short read panels represent the reads mapped with proper insert size and not. The grey lines in long read panels represent read alignments and links between primary and supplementary alignments of the same read. The read mapping patterns were drawn using IGV program.


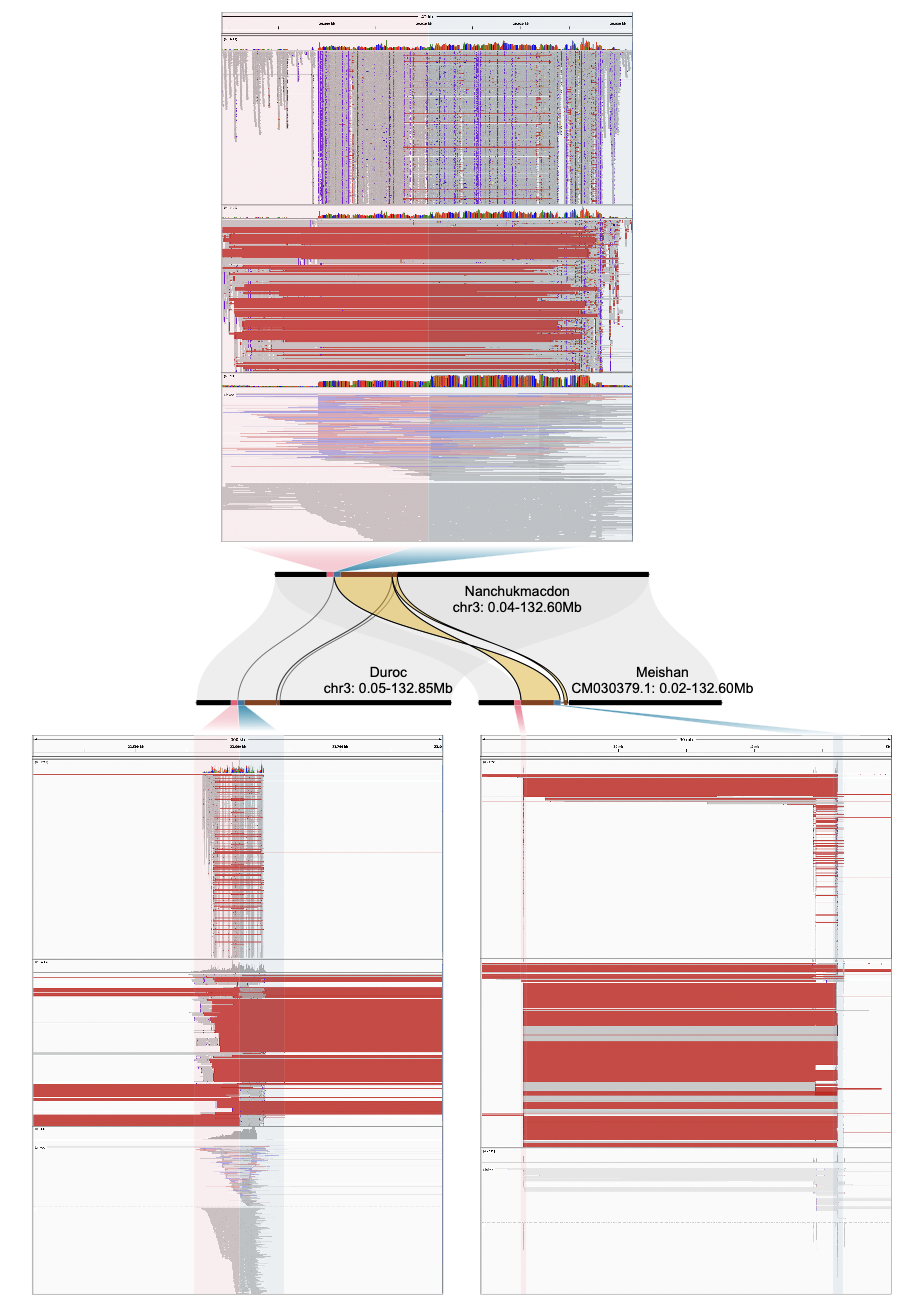


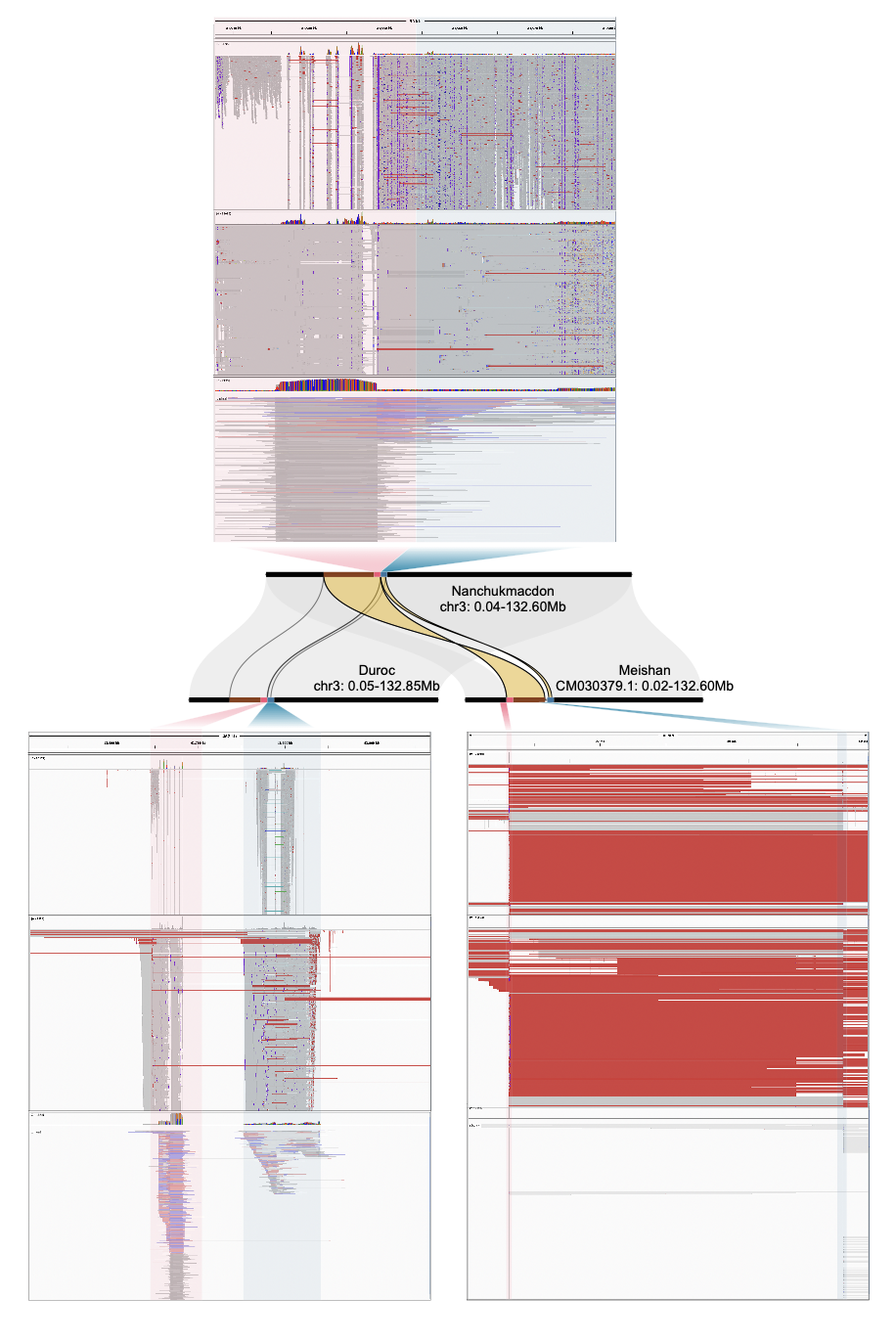


**
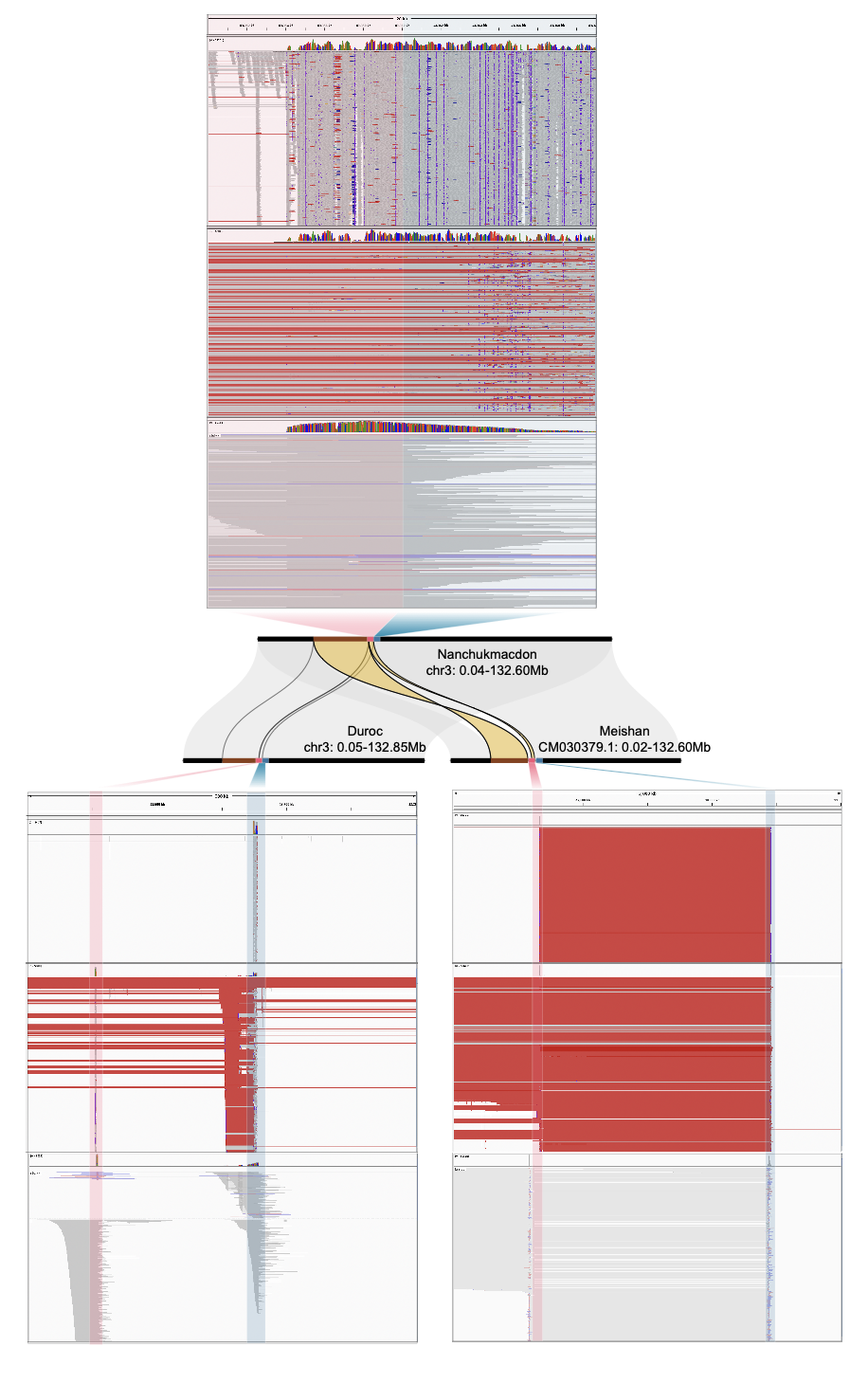
**

**Figure S3.** Mapping patterns of Nanchukmacdon reads at the breakpoint regions of different pig breed assemblies in chromosome 3. The panels show the mapping depths and patterns of paired-end (Top), mate pair (Middle), and long reads (Bottom) at the breakpoint regions of each breed, respectively. The reads which were mapped with mapping quality larger than 10 were only shown in the figure. The grey and red lines in short read panels represent the reads mapped with proper insert size and not. The grey lines in long read panels represent read alignments and links between primary and supplementary alignments of the same read. The read mapping patterns were drawn using IGV program.

**
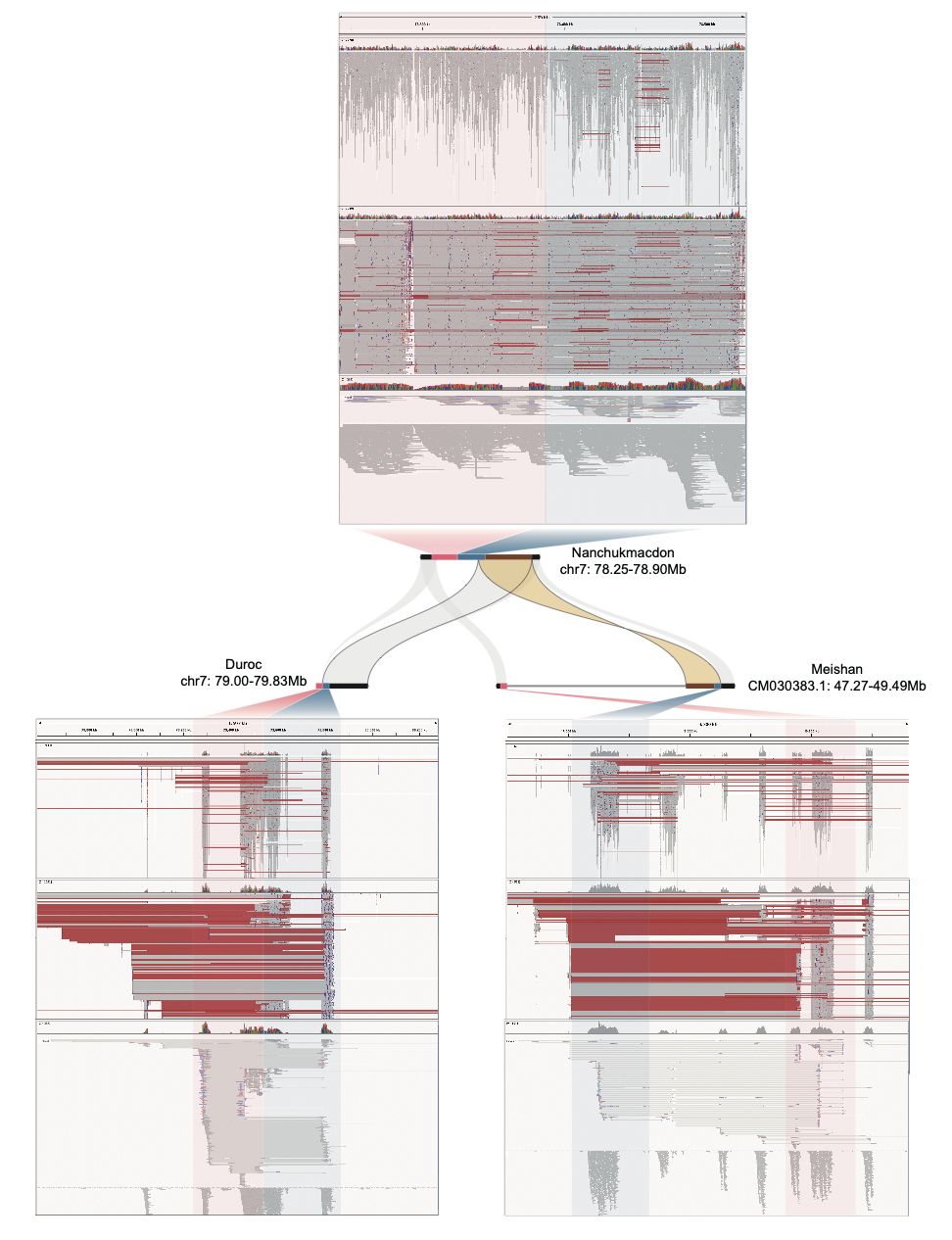

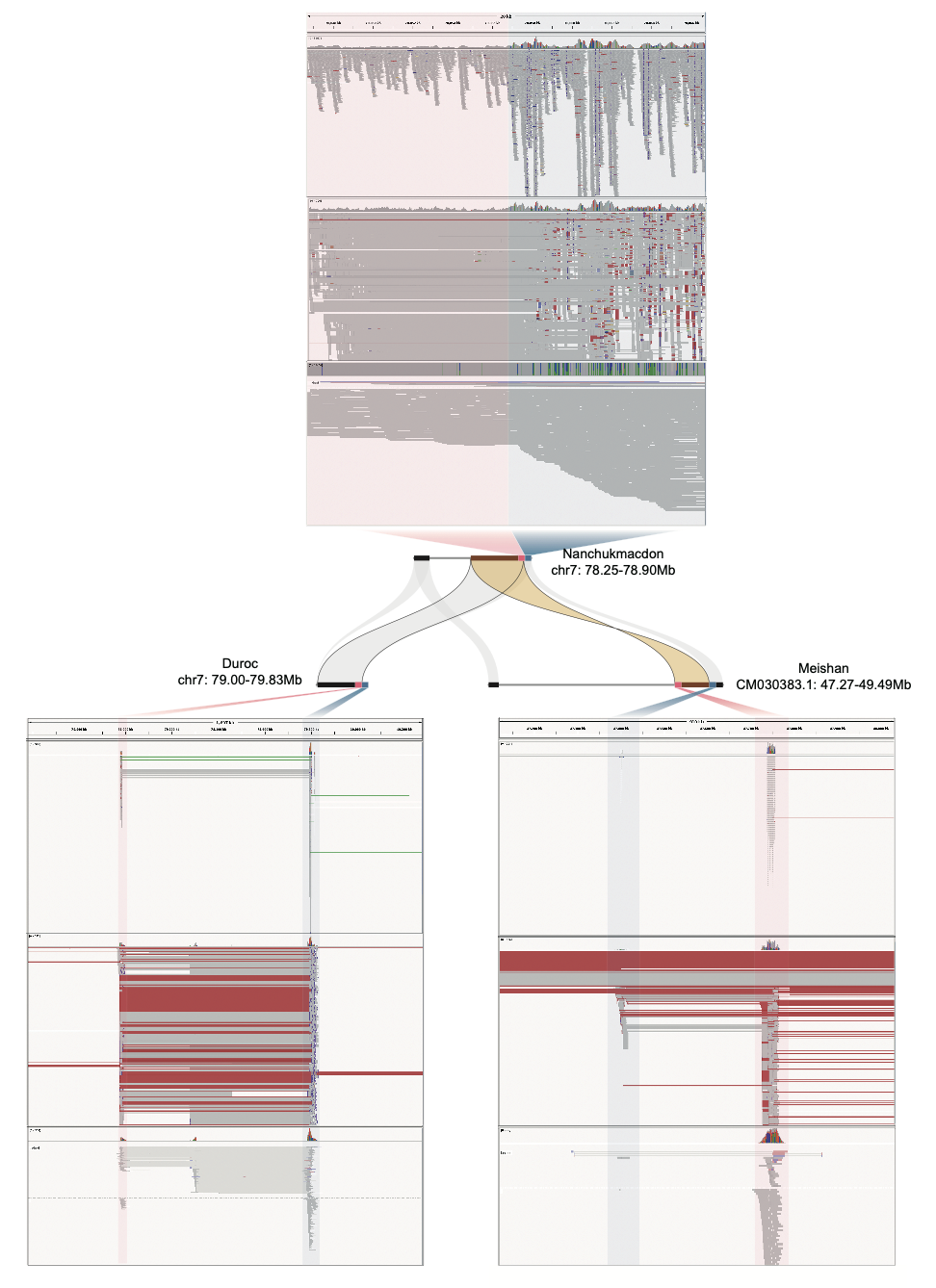
**

**Figure S4.** Mapping patterns of Nanchukmacdon reads at the breakpoint regions of different pig breed assemblies in chromosome 7. The panels show the mapping depths and patterns of paired-end (Top), mate pair (Middle), and long reads (Bottom) at the breakpoint regions of each breed, respectively. The reads which were mapped with mapping quality larger than 10 were only shown in the figure. The grey and red lines in short read panels represent the reads mapped with proper insert size and not. The grey lines in long read panels represent read alignments and links between primary and supplementary alignments of the same read. The read mapping patterns were drawn using IGV program.


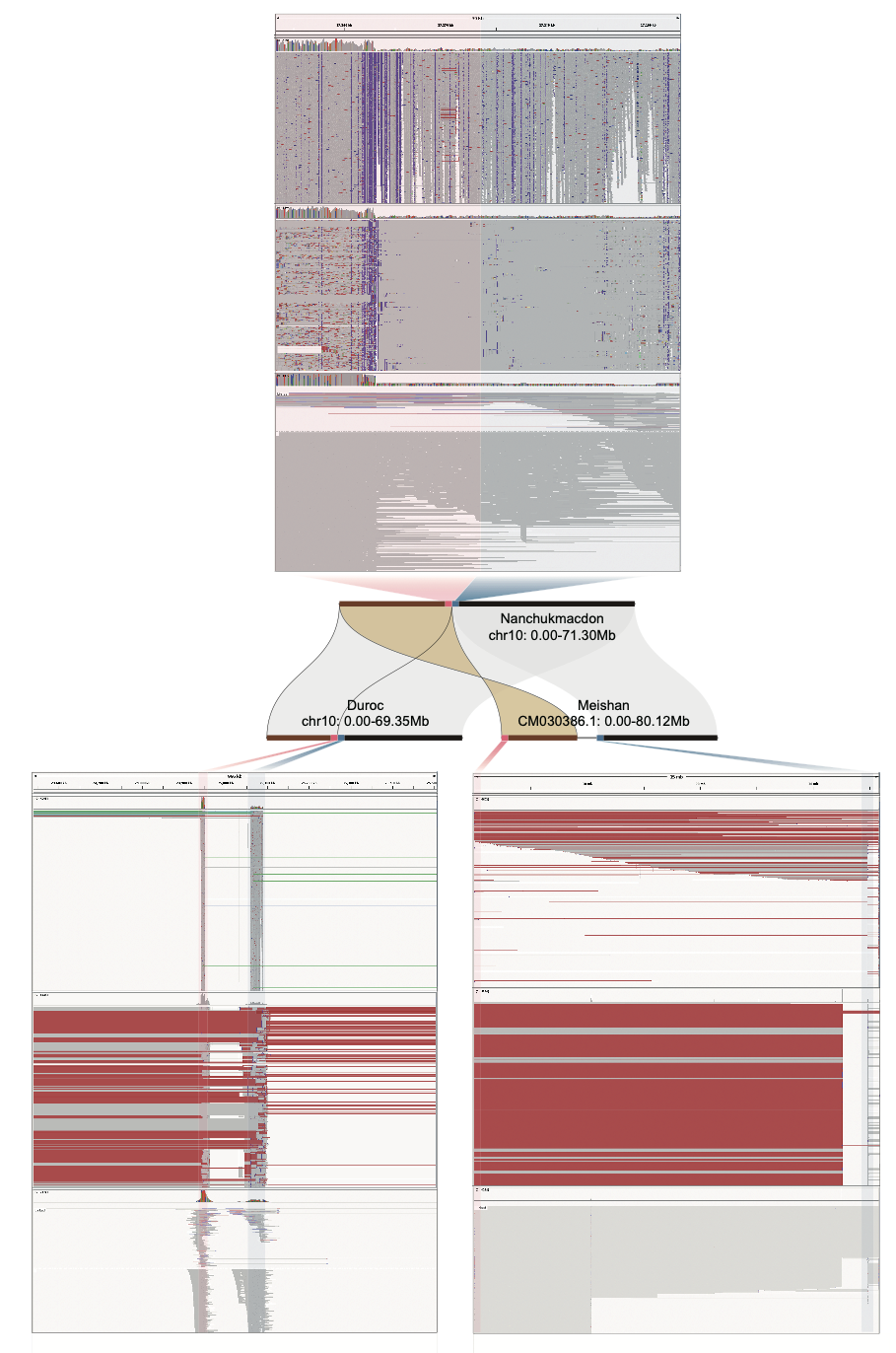


**Figure S5.** Mapping patterns of Nanchukmacdon reads at the breakpoint regions of different pig breed assemblies in chromosome 10. The panels show the mapping depths and patterns of paired-end (Top), mate pair (Middle), and long reads (Bottom) at the breakpoint regions of each breed, respectively. The reads which were mapped with mapping quality larger than 10 were only shown in the figure. The grey and red lines in short read panels represent the reads mapped with proper insert size and not. The grey lines in long read panels represent the read alignments and links between primary and supplementary alignments of the same read. The read mapping patterns were drawn using IGV program.

**
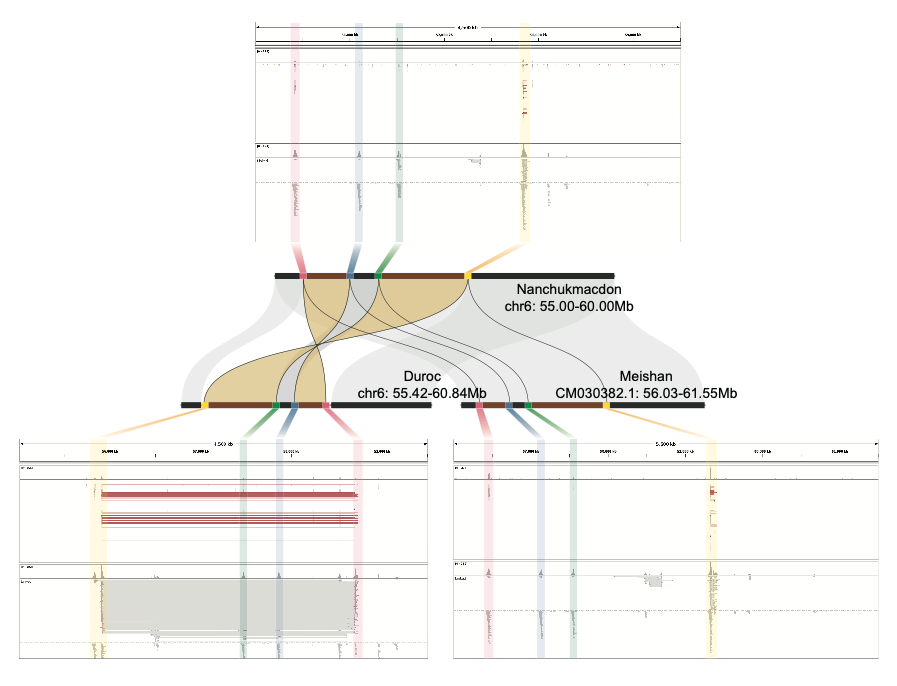

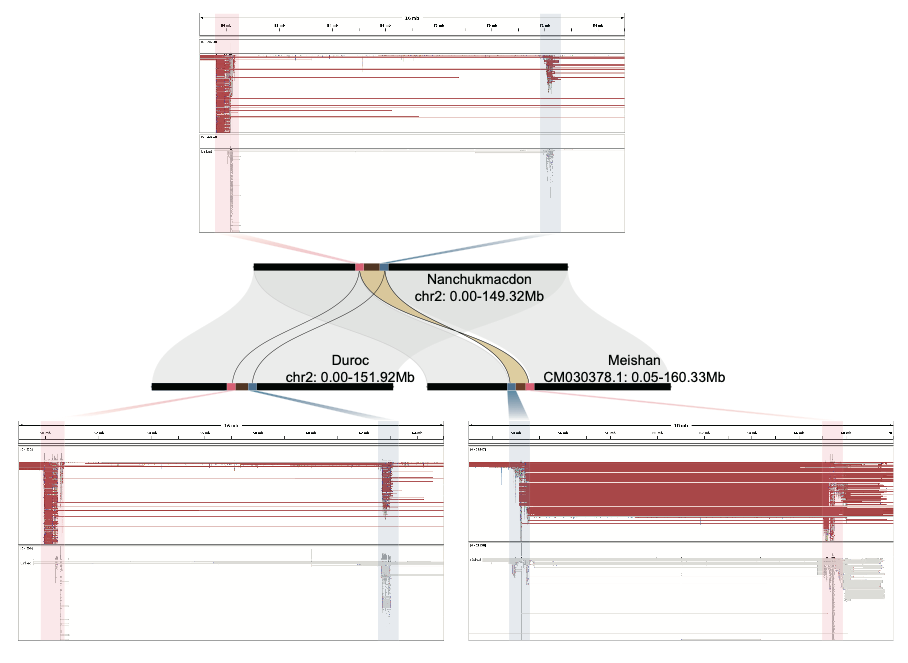

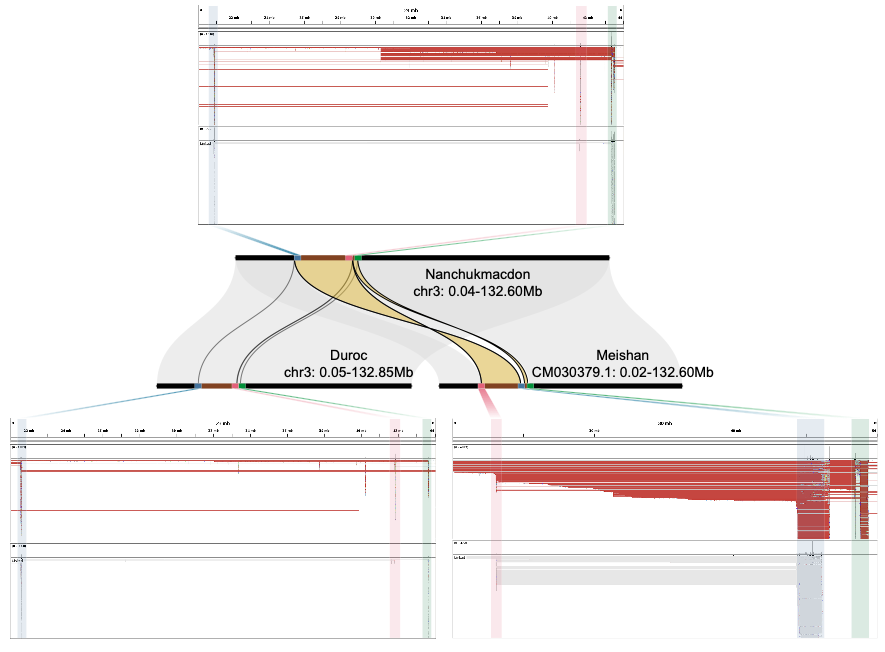

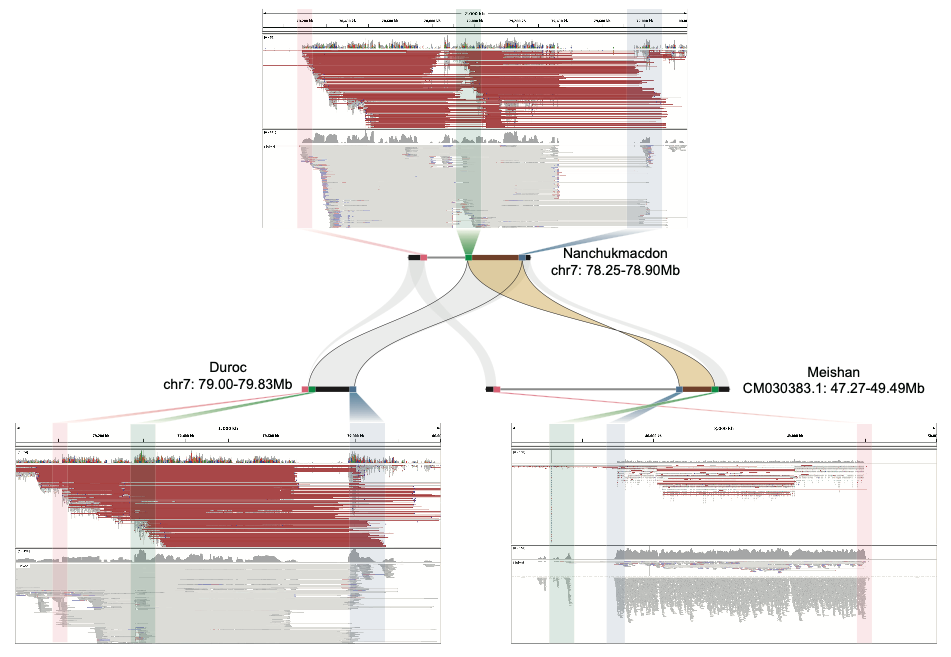

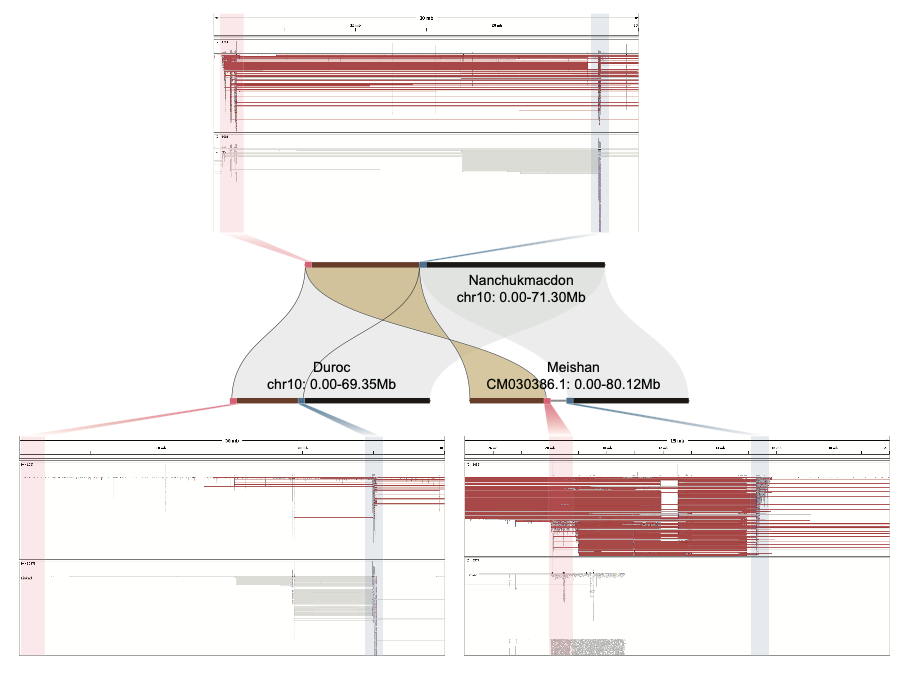
**

**Figure S6.** Mapping patterns of Meishan reads at the breakpoint regions of different pig breed assemblies. The panels show the mapping depths and patterns of short reads (Top) and long reads (Bottom) at the breakpoint regions of each breed, respectively. The reads which were mapped with mapping quality larger than 10 were only shown in the figure. The grey and red lines in short read panels represent the reads mapped with proper insert size and not. The grey lines in long read panels represent read alignments and links between primary and supplementary alignments of the same read. The read mapping patterns were drawn using IGV program.


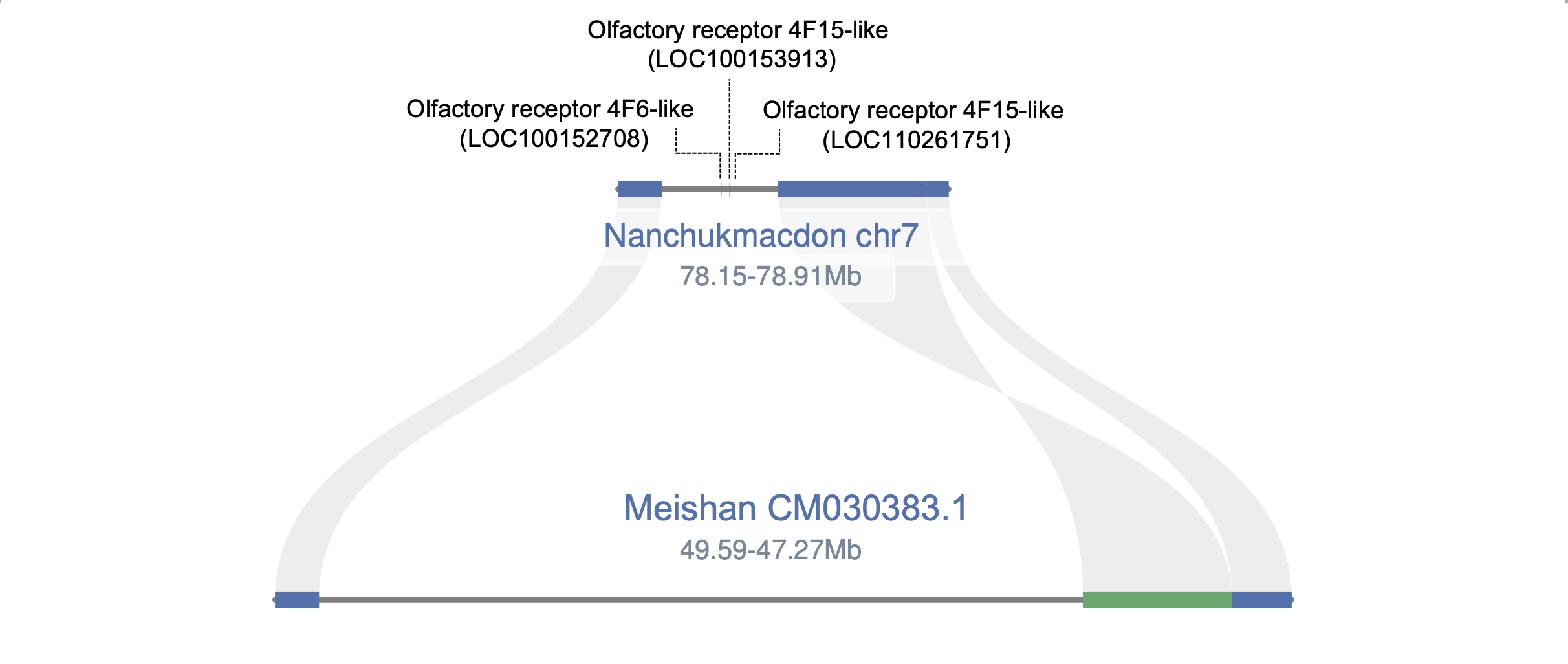


**Figure S7.** Paralogous genes located in breakpoint regions at the Nanchukmacdon chromosome 7.

**Figure S8.** PCA result of 29 samples of three different pig breeds based on NSVs with minor allele frequency not lower than 0.05 and missing rate not larger than 0.1.
